# Promotion

**Published:** 1862-10

**Authors:** 


### PROMOTION.

We understand an effort is being made for the promotion of our worthy friend, Col. John T. Toland, of the 34th Regiment O. V. I., to the position of Brigadier General. This we should think an appointment eminently fit to be made. The Colonel is a brave and efficient officer—one who knows his duty, and under all circumstances has the boldness and courage to perform it. This movement will, doubtless, give unfeigned pleasure to all who know him. T.

